# Effects of saline drinking water on growth performance, carcass traits, and blood biochemistry in crossbred Muscovy ducks

**DOI:** 10.14202/vetworld.2025.782-790

**Published:** 2025-04-07

**Authors:** Le Thanh Phuong, Nguyen Thi Thuy, Nguyen Thiet

**Affiliations:** 1 Emivest Feedmill Vietnam Company Limited, Binh Duong, 820000, Vietnam; 2 Faculty of Animal Sciences, College of Agriculture, Can Tho University, Can Tho, 900000, Vietnam; 3 Department of Agricultural Technology, College of Rural Development, Can Tho University, Can Tho, 900000, Vietnam

**Keywords:** blood biochemistry, carcass traits, Muscovy ducks, saline water, salt gland, water salinity

## Abstract

**Background and Aim:**

Water quality is a critical factor in duck farming, influencing growth performance, health, and productivity. Salinity in drinking water is a concern in regions affected by saline intrusion, such as the Mekong Delta, Vietnam. Notably, female ducks exhibit greater salt tolerance than males due to differences in kidney size and function. This study aimed to investigate the effects of saline drinking water on the growth performance, carcass traits, and blood biochemistry of crossbred Muscovy ducks (*Cairina moschata*).

**Materials and Methods:**

A completely randomized design was used, including five treatments with five replications per treatment. Each replication consisted of four crossbred Muscovy ducks, with an equal number of males and females. The treatments comprised freshwater (SW0.0) and diluted seawater with salinity levels of 0.15% (SW0.15), 0.3% (SW0.3), 0.45% (SW0.45), and 0.6% (SW0.6). Ducks were provided *ad libitum* access to feed and water throughout the experiment. Key parameters measured included dry matter intake (DMI), water intake, body weight (BW), carcass traits, meat quality, salt gland dimensions, and blood biochemical parameters.

**Results:**

Saline drinking water significantly reduced DMI and BW gain, with male ducks more adversely affected than females. The final BW of males decreased with increasing salinity, while female ducks maintained stable BWs. Ducks consuming water with salinity levels of 0.45% and 0.6% had higher feed conversion ratios (4.83 and 4.68, respectively) compared to lower salinity groups (3.77–4.61). Carcass weight, breast weight, and abdominal fat percentage declined with increasing salinity, whereas thigh and drumstick weights remained unaffected. The crude protein content of thigh and drumstick meat decreased at higher salinity levels. Blood biochemical parameters, including sodium, chloride, urea, and creatinine levels, increased in ducks consuming highly saline water, indicating potential physiological stress. Ducks in higher salinity groups exhibited increased salt gland size, suggesting an adaptive response to saline conditions.

**Conclusion:**

Male crossbred Muscovy ducks can tolerate drinking water salinity levels up to 0.3%, whereas females can withstand salinity up to 0.6%. The greater salinity tolerance of female ducks makes them more suitable for farming in saline-affected areas. While increased salt gland size suggests an adaptive response, elevated blood biochemical markers indicate potential physiological stress. Future research should explore strategies to mitigate the negative effects of saline water on duck production, including dietary interventions with antioxidants such as vitamin E or selenium.

## INTRODUCTION

Ducks are the second most important poultry species after chickens in terms of egg and meat production. Asian countries account for approximately 80% of global duck meat production. Muscovy ducks are particularly well-suited to scavenging systems and exhibit greater adaptability to hot climates than chickens [1]. Water quality is a critical factor in duck farming because ducks rely on water for drinking, feeding, and bathing. Poor water quality can have harmful effects on duck health, leading to increased susceptibility to diseases, reduced feed intake, and impaired growth rates. Krista *et al*. [2] demonstrated that exposure to saline water containing 4000 parts per million (ppm) sodium chloride (NaCl) negatively affects duck growth and feed utilization efficiency, leading to reduced productivity and profitability for farmers. In addition, Tulu *et al*. [3] suggested that poultry consuming saline water varying from 2.35 g/L to 3.15 g/L was associated with loss of production and health impairment. However, Sulaiman *et al*. [4] found that Alabio ducks consuming saline water containing 3 g/L (0.3%) of total dissolved solids (TDS) showed no significant effect on egg production. In short, these findings suggest that saline water at a concentration of 0.3% is safe for ducks and broilers.

In the coastal regions of the Mekong Delta in Vietnam, saline water intrusion is a prevalent environmental issue intensified by factors such as climate change, land use practices, and improper water management. Anh [5] reported that during the dry season, coastal provinces of the Mekong Delta are affected by saline intrusion, with salt concentrations exceeding 4 g/L. *Cairina moschata* (Muscovy duck) farming in the Mekong Delta has long been a significant agricultural activity, contributing to both rural livelihoods and the country’s poultry industry. Muscovy ducks are valued for their relatively low maintenance requirements, adaptability to diverse environments, and quality of their meat, which is considered lean and flavorful [6]. The salinity levels in water bodies used for duck farming can fluctuate significantly, posing challenges for farmers and potentially impacting Muscovy duck production.

Despite the crucial role of water quality in poultry farming, limited research has specifically addressed the impact of saline drinking water on the growth performance, carcass traits, and blood biochemical responses of Muscovy ducks. Existing studies have primarily focused on broilers and other waterfowl species, with little emphasis on sex-based differences in salinity tolerance among crossbred Muscovy ducks. Furthermore, the physiological mechanisms underlying salinity adaptation, particularly in terms of salt gland development and metabolic responses, remain underexplored.

This study aims to evaluate the effects of saline drinking water on growth performance, carcass traits, blood biochemical parameters, and salt gland characteristics in crossbred Muscovy ducks, with a specific focus on sex-based differences in salinity tolerance. Both sexes were included in this study because female and male ducks may differ in saline tolerance, as previously reported by Hughes *et al*. [7]. The findings will provide insights into the adaptability of Muscovy ducks to saline conditions, offering practical recommendations for poultry management in regions affected by saline water intrusion.

## MATERIALS AND METHODS

### Ethical approval

The study was carried out in accordance with the guidelines laid down by the International Animal Ethics Committee and was approved by the Animal Experimental Council of Can Tho University (number CTU-AEC24015).

### Study period and location

The study was conducted from March to May 2024 at the Experimental Farm, College of Rural Development, Can Tho University. The air temperature and relative humidity percentages at 09:00 and 13:00 were 31.25 ± 0.12 and 35.50 ± 0.24°C and 49.00 ± 1.53 and 32.25 ± 0.43%, respectively.

### Experimental management and design

A total of 100 crossbred Muscovy ducks, approximately 21 days old, were obtained from local farmers and vaccinated following the farm’s protocol. The ducks were housed in pens measuring 1.0 × 3.0 m with plastic flooring in an open-sided housing system. Each pen was equipped with a feeder and a 5-L drinking container, providing *ad libitum* access to feed and water throughout the experiment.

The experiment followed a completely randomized design, consisting of five treatments with five replications per treatment. Each replication included four crossbred Muscovy ducks, ensuring an equal distribution of males and females. The treatments comprised ducks consuming freshwater (SW0.0) and those consuming diluted seawater at varying salinity levels: 0.15% (SW0.15), 0.3% (SW0.3), 0.45% (SW0.45), and 0.6% (SW0.6). The saline water for the treatment groups was prepared by diluting concentrated seawater (9%) sourced from aquaculture farms with freshwater. The final saline concentrations were achieved using the dilution formula: C_1_V_1_ = C_2_V_2_, where C_2_ and V_2_ represent the concentration and volume of the initial solution, while C_2_ and V_2_ correspond to the desired concentration and volume of the final solution. The salinity levels of the prepared solutions were verified using a water salinity meter. The composition of both freshwater and concentrated seawater had been previously reported in the literature.

The experiment lasted for 9 weeks, including a 1-week adaptation period followed by 8 weeks of data collection. Ducks were fed a commercial meat duck concentrate twice daily at 08:00 and 14:00 h. The feed had a crude protein (CP) content of 18% and a metabolizable energy value of 3000 kcal/kg. The NaCl content of the feed was not analyzed in this study.

### Data collection and measurement

Feed and water intake (WI) were collected weekly throughout the experiment. Feed offers and refusals were collected once a week, stored in the freezer, and then mixed at the end of the experiment for dry matter (DM) analysis. DM was determined using the Association of Official Analytical Chemists (AOAC) method [8].

The body weights (BWs) of all ducks were determined at the beginning and end of the experiment. The ducks were weighed in morning feeding using an electronic scale with an accuracy of ±0.05 g.

### Parameters for slaughter and meat quality

#### Animal preparation and slaughter parameters

At the end of the experiment, four ducks per group, with the average weight of each group, were selected to evaluate meat quality and salt gland dissection. The ducks were slaughtered under similar conditions to minimize any other factors influencing the meat qualities and according to the procedures presented in a previous study by Zhang *et al*. [9]. Briefly, feed was withdrawn 12 h before slaughter, and ducks were sacrificed by neck cutting, scalded with hot water (60°C for 3 min), and feathers were removed mechanically. The slaughter parameters were carcass weight, thigh and drumstick ratio, breast ratio, and abdominal fat ratio.

#### Measurement of meat quality

Chemical composition of meat: Breast, thigh, and drumstick meat samples were taken from each treatment from 20 individuals (4 ducks per treatment) for chemical composition analysis at the time of slaughter. The parameters included DM content (%), CP content (%), crude fat content (%), and total mineral content (%) according to the AOAC method [8].

Meat pH and color were assessed in the left breast, thigh, and drumstick muscles. Muscle lightness (L*), redness (a*), and yellowness (b*) were determined using a chroma meter (Konica Minolta, Tokyo, Japan). A pH meter (HI991001, Hanna Instruments, Romania) was used to record the pH at 0 h (pH_0_) and 24 h (pH_24_, muscle samples were stored for 24 h at 4°C) after slaughtering.

### Dissection of the salt gland

The salt gland was dissected according to the recommendation of Anh [10] with the following steps: (1) Removed the feathers, skin, and surrounding tissues around the eye region; (2) exposed the salt gland located in the groove of the upper rim of the eye socket in the frontal and lacrimal bone regions; and (3) used a sharp-pointed #11 scalpel to separate the gland along the curve of the eye socket to detach it from the groove, then waited for about 1 min for the tissues around the gland to dry after dissection. Parameters were determined, including the length of the gland (the distance between the two ends of the gland) and the width of the gland (at its widest point) measured with a ruler (with the smallest division in millimeters). The weight of the gland was measured using a scale that was accurate to milligrams.

### Blood biochemical parameters

At the end of the experiment, blood samples were collected from 4 ducks per treatment at the same time of slaughter. Each duck was collected 2 mL of blood into heparin tubes, kept in crushed ice, and brought to the laboratory for centrifugation to harvest the plasma and then kept in the freezer for later analysis. The blood samples were analyzed for electrolytes (sodium, potassium, chloride, and calcium), urea, creatinine, aspartate aminotransferase (AST), and alanine aminotransferase (ALT). An automated clinical chemistry analyzer was used to measure the concentrations of urea, creatinine, AST, and ALT in plasma (XL200, Erba Mannheim, Germany) and electrolytes in plasma (ST200 PRO, Sensa Core, India).

### Statistical analysis

All data were analyzed using one-way analysis of variance to determine the effects of different salinity levels in drinking water on growth performance, carcass traits, meat quality, blood biochemical parameters, and salt gland characteristics of crossbred Muscovy ducks. Before analysis, data were checked for normality and homogeneity of variance using the Shapiro–Wilk test and Levene’s test, respectively.

When significant differences were detected, Tukey’s *post hoc* test was performed for pairwise comparisons among treatments. Results were expressed as means ± standard error of the mean. A significance level of p < 0.05 was used to determine statistical differences among treatments.

All statistical analyses were conducted using the Minitab Statistical Software, version 16 (Minitab Incorporation, State College, PA, USA).

## RESULTS

### Effects of saline water on DM and WI

There was a significant effect of salinity in drinking water on DMI and WI (Table 1; p < 0.05). As the salinity of water increases, the DMI decreases and the WI increases. However, ducks that consumed either high (0.6%) or low (0.15%) saline water had similar WI as those in the freshwater group (Table 1; p < 0.05).

**Table 1:** Effects of saline water on the average DMI and WI, BW, weight gain, and FCR of crossbred Muscovy ducks.

Items	Treatments					SEM	p-value

	SW0.0	SW0.15	SW0.3	SW0.45	SW0.6		
Average DMI (g/head/day)	115.06^a^	117.94^a^	109.29^b^	108.95^b^	97.68^b^	3.27	0.001
Average daily WI (g/head/day)	556.2^c^	536.8^c^	748.6^ab^	827.8^a^	605.5^bc^	36.94	0.001
Initial BW (g/head)	710.00	680.00	688.75	737.50	710.00	20.64	0.35
Final BW (g/head)	2426.25^a^	2239.58^a^	2143.75^ab^	2087.50^ab^	1882.50^b^	85.45	0.01
Final male BW (g/head)	3080^a^	3100^a^	2675^b^	2638^b^	2225^c^	96.25	0.001
Final female BW (g/head)	1878	1796	1883	1796	1646	64.02	0.101
Weight gain (g/head/day)	30.65^a^	27.85^ab^	25.98^bc^	24.11^bc^	20.94^c^	1.56	0.01
FCR	3.77^b^	4.61^ab^	4.22^ab^	4.83^a^	4.68^a^	0.22	0.02

^a,b,c^Values with different superscripts within the same row differ (p < 0.05). SW0.0=Ducks consuming fresh water, SW0.15=Ducks consuming diluted seawater at 0.15%, SW0.30=Ducks consuming diluted seawater at 0.3%, SW0.45=Ducks consuming diluted seawater at 0.45%, SW0.6=Ducks consuming diluted seawater at 0.6%, FCR=Feed consumption ratio, DMI=Dry matter intake, WI=Water intake, SEM=Standard error of the mean, BW=Body weight

### Effects of saline water on BW, BW gain (BWG), and feed conversion ratio (FCR)

Final BW was negatively affected by saline water in male ducks but remained unchanged in female ducks. The weight gain decreased and FCR increased as the salinity of the water increased (Table 1; p < 0.05).

### Effects of saline water on the carcass traits of crossbred Muscovy ducks

The effects of saline water on the carcass traits of crossbred Muscovy ducks were significant. The percentages of carcass weight, breast weight, and abdominal fat decreased as the ducks consumed them with gradually increasing salinity (Table 2; p < 0.05).

**Table 2:** Effects of saline water on the carcass traits of crossbred Muscovy ducks.

Traits	Treatments					SEM	p-value

	SW0.0	SW0.15	SW0.3	SW0.45	SW0.6		
Carcass weight, % BW	78.92^a^	75.81^ab^	74.85^b^	75.01^b^	74.83^b^	0.93	0.02
Breast weight, % carcass weight	18.71^a^	19.19^a^	15.87^b^	15.75^b^	15.48^b^	0.64	0.001
Thigh and drumstick weight, % carcass weight	11.29	10.84	10.85	10.14	11.17	0.30	0.10
Abdominal fat percentage, carcass weight	1.17^a^	0.91^a^	0.59^b^	0.39^b^	0.29^b^	0.10	0.001

^a,b^Values with different superscripts within the same row differ (p < 0.05). SW0.0=Ducks consuming fresh water, SW0.15=Ducks consuming diluted seawater at 0.15%, SW0.30=Ducks consuming diluted seawater at 0.3%, SW0.45=Ducks consuming diluted seawater at 0.45%, SW0.6=Ducks consuming diluted seawater at 0.6%, SEM=Standard error of the mean, BW=Body weight

### Effects of saline water on the color and chemical composition of breast, thigh, and drumstick meat and plasma biochemical parameters of crossbred Muscovy ducks

There were no effects of saline water on the color and chemical composition of breast meat in crossbred Muscovy duck (Table 3; p < 0.05). However, the pH values of thigh and drumstick meat in SW0.45 at 0 h and 24 h were higher than those of the other treatments. In addition, the CP content of thigh and drumstick meat decreased as the salinity of the water increased (Table 3; p < 0.05).

**Table 3:** Effects of saline water on the pH, color, and chemical composition of the breast, thigh, and drumstick meat of crossbred Muscovy ducks.

Items	Treatments					SEM	p-value

	SW0.0	SW0.15	SW0.3	SW0.45	SW0.6		
Breast							
pH-0h	5.90	5.89	5.84	5.93	5.81	0.03	0.08
pH-24h	5.85	5.73	5.65	5.78	5.72	0.06	0.19
L*	48.24	47.19	47.07	48.26	45.98	1.94	0.91
a*	13.08	14.75	14.59	13.96	13.66	1.34	0.90
b*	5.15	5.02	5.30	5.55	6.02	0.55	0.96
DM (%)	26.55	25.87	26.27	25.10	25.90	0.47	0.29
CP (%)	17.67	17.99	17.92	17.61	17.94	1.70	0.22
EE (%)	5.13	5.44	5.47	5.85	6.13	0.35	0.33
Ash (%)	2.15	1.78	1.91	1.72	1.70	0.22	0.58
Thigh and drumstick motion							
pH-0h	6.35^ab^	6.13^c^	6.21^bc^	6.48^a^	6.18^bc^	0.04	0.001
pH-24h	6.15^ab^	6.07^b^	6.09^b^	6.39^a^	5.99^b^	0.06	0.001
L*	48.76	51.73	52.56	50.01	51.88	1.73	0.53
a*	17.13	15.69	17.04	15.77	15.78	1.72	0.94
b*	5.90	5.42	6.78	6.01	6.45	0.58	0.53
DM (%)	26.12	26.82	26.02	25.12	26.57	0.80	0.61
CP (%)	19.00^a^	18.27^ab^	16.97^b^	17.57^ab^	15.52^b^	0.38	0.001
EE (%)	4.70	4.84	4.07	3.82	5.00	0.76	0.77
Ash (%)	1.13	1.01	0.95	0.90	1.16	0.08	0.17

^a,b,c^Values with different superscripts within the same row differ (p < 0.05). SW0.0=Ducks consuming fresh water, SW0.15=Ducks consuming diluted seawater at 0.15%, SW0.30=Ducks consuming diluted seawater at 0.3%, SW0.45=Ducks consuming diluted seawater at 0.45%, SW0.6=Ducks consuming diluted seawater at 0.6%, DM=Dry matter, CP=Crude protein, EE=Ether extract, SEM=Standard error of the mean, L*=Lightness, a*=Redness, b*=Yellowness

Ducks consuming saline water had increased plasma levels of sodium and chloride (Table 4; p < 0.05). However, plasma potassium and calcium levels remained unchanged among the treatments in this study. The plasma levels of urea, creatinine, and AST increased with higher saline water concentrations (Table 4; p < 0.05), whereas saline water did not affect the ALT concentration.

**Table 4:** Effects of saline water on plasma biochemical parameters of crossbred Muscovy ducks.

Items	Treatments					SEM	p-value

	SW0.0	SW0.15	SW0.3	SW0.45	SW0.6		
Na (mmol/L)	139.63^b^	140.25^b^	142.73^a^	141.95^a^	142.23^a^	0.39	0.001
K (mmol/L)	5.62	5.90	5.57	5.91	5.51	0.20	0.51
Cl (mmol/L)	102.40^b^	103.55^b^	104.90^a^	104.85^a^	105.77^a^	0.41	0.001
Ca (mmol/L)	1.26	1.25	1.27	1.29	1.28	0.02	0.50
Urea (mmol/L)	0.332^b^	0.382^b^	0.386^b^	0.610^a^	0.410^a^	0.03	0.001
Creatinine (µmol/L)	18.50^b^	19.68^b^	21.40^ab^	24.18^a^	21.00^ab^	0.87	0.01
AST (U/L)	25.43^b^	37.90^b^	42.45^ab^	58.30^a^	32.83^b^	4.87	0.01
ALT (U/L)	8.88	7.63	10.20	11.10	10.20	0.86	0.08

^a,b^Values with different superscripts within the same row differ (p < 0.05). SW0.0=Ducks consuming fresh water, SW0.15=Ducks consuming diluted seawater at 0.15%, SW0.30=Ducks consuming diluted seawater at 0.3%, SW0.45=Ducks consuming diluted seawater at 0.45%, SW0.6=Ducks consuming diluted seawater at 0.6%, Na=Sodium, K=Potassium, Cl=Chloride, Ca=Calcium, AST=Aspartate aminotransferase, ALT=Alanine aminotransferase, SEM=Standard error of the mean

### Effects of saline water on the salt gland characteristics of crossbred Muscovy ducks

There was a significant effect of saline water on the salt gland characteristics of crossbred Muscovy ducks (Table 5; p < 0.05). Ducks consuming saline water had significantly greater length, width, and weight of salt glands compared to those in the freshwater group.

**Table 5:** Effects of saline water on the salt gland characteristics of crossbred Muscovy ducks.

Items	Treatments					SEM	p-value

	SW0.0	SW0.15	SW0.3	SW0.45	SW0.6		
Length, mm	24.55^b^	28.88^ab^	29.60^a^	33.83^a^	31.85^a^	1.24	0.001
Wide, mm	6.55^b^	7.08^b^	9.43^a^	8.90^ab^	9.28^a^	0.46	0.001
Weight, g	0.212^b^	0.390^a^	0.386^a^	0.432^a^	0.442^a^	0.03	0.001

^a,b^Values with different superscripts within the same row differ (p < 0.05). SW0.0=Ducks consuming fresh water, SW0.15=Ducks consuming diluted seawater at 0.15%, SW0.30=Ducks consuming diluted seawater at 0.3%, SW0.45=Ducks consuming diluted seawater at 0.45%, SW0.6=Ducks consuming diluted seawater at 0.6%, SEM=Standard error of the mean

## DISCUSSION

Although crossbred Muscovy ducks can tolerate salinity levels of up to 0.3%. However, there were no dead ducks among the treatments throughout the experiment.

### Effects of saline water on DM and WI

Ducks that consumed saline water at concentrations between 0.3% and 0.6% exhibited decreased DMI, whereas ducks that consumed 0.15% saline water maintained similar DMI levels compared to the freshwater group. The reduced DMI in the saline groups may be attributed to the water-filled crops of birds [2, 11] or the high salt concentration, which could decrease their appetite. This decrease in appetite may be due to lesions in the appetite center of the lateral nucleus of the hypothalamus or inflammation in the rectum of birds [12]. Our observations revealed that ducks consuming high-saline water had higher water content in their feces than those in the control group. In addition, ducks in the high-saline groups appeared uncomfortable and spent more time standing, with poor feathering, than those in the control group.

WI increased gradually with salt concentrations ranging from 0% to 0.45% but then decreased at a concentration of 0.6%. This decrease in WI may be due to the extremely salty flavor, which has a limiting effect on intake, as noted previously by Odoi *et al*. [13]. When birds drink saline water, the osmolality of their body fluids increases, prompting them to minimize osmotic stress by reducing their salt intake [14]. Similarly, Chandrashekar *et al*. [15] observed that low levels of saline water stimulated drinking behavior in mice, but at higher salt concentrations, the mice avoided drinking to reduce salt stress. Sulaiman *et al*. [4] found that Alabio ducks consuming saline water with 3 g/L of TDS experienced no significant effects on DMI or WI. In this study, ducks consuming 3 g/L of TDS showed an increase in WI but a subsequent decrease in DMI. High sodium concentrations have been shown to increase DMI in broiler chicks [16] and Japanese quails [11]. Krista *et al*. [2] observed that ducklings consuming 4000 ppm of added NaCl exhibited increased WI and loss of appetite. In summary, these findings suggest that saline water at a concentration of 3% is safe for ducks and broilers.

### Effects of saline water on BW, BWG, and FCR

Muscovy ducks consuming water with a 0.6% saline concentration exhibited decreased final BW, BWG, and an increased FCR compared to those drinking fresh water. The reduced performance of these ducks may be attributed to their lower DM intake (DMI) and increased osmotic stress [17]. Contrary to these findings, Khalilipour *et al*. [11] observed that Japanese quails drinking high-saline water showed increased DMI, although BW and BWG decreased. Similarly, Ahmed [18] found that broiler chicks consuming saline water with up to 2610 ppm of TDSs experienced no negative impacts on growth performance, whereas higher concentrations led to a decrease in growth rate. In line with this, Krista *et al*. [2] reported growth retardation in ducklings that consumed water containing 4000 ppm NaCl. In this study, saline water was found to affect BW in male Muscovy ducks but not in females. This response is consistent with the findings of Hughes *et al*. [7], who observed that female Pekin ducks were more tolerant to salt than their male. The better saline water tolerance of female ducks may be due to their larger kidney size and higher glomerular filtration rate than those of male ducks [19, 20].

Our study indicated that Muscovy ducks consu-ming saline water at concentrations between 0.45% and 0.6% had higher FCR than those consuming lower saline water levels (0.15% and 0.3%) or freshwater. Consistent with these findings, other studies have shown that FCR and overall performance decreased as water salinity increased in broiler chicks [16] and Japanese quails [11]. The increase in FCR and performance may be due to an imbalance in electrolytes, which can affect various physiological and metabolic functions [16]. However, Sulaiman *et al*. [4] found that Alabio ducks consuming saline water supplemented with 3 g/L of TDS showed no significant effect on egg production.

### Effects of saline water on the carcass traits of crossbred Muscovy ducks

Carcass weight, breast weight, and abdominal fat were affected by saline water levels ranging from 0.3% to 0.6%, whereas thigh and drumstick weights remained consistent across groups. The observed reduction in carcass traits in crossbred Muscovy ducks may be due to lower feed consumption. Contrary to these findings, De Oliveira Carvalho *et al*. [21] reported that slow-growing chickens consuming low saline water (<3 g/L of TDS) had increased DMI and carcass weight. However, in marine ducks (*Anas platyrhynchos*), Hughes *et al*. [22] found that breast muscle mass was lower in the freshwater group compared to the saline group (300 mM NaCl), with no differences in feed intake between groups. Carcass weight in this study ranged from 74.83% to 78.92%, which is higher than the range reported by Marakan *et al*. [23], who observed carcass weights in Muscovy ducks varying from 68.5% to 71.6%. However, the breast yield in this study was lower than that reported by Costa *et al*. [24]. No effect of saline water on thigh and drumstick weight was observed in this study, which is consistent with findings of De Oliveira Carvalho *et al*. [21]. However, abdominal fat decreased as saline water levels increased because of reduced energy intake or increased energy expenditure for excreting excess salts. The decrease in abdominal fat in the saline water groups contrasts with the findings of De Oliveira Carvalho *et al*. [21] in chickens. This difference may be due to the higher subcutaneous fat content in duck carcasses, as noted by Kokoszyński *et al*. [25].

### Effects of saline water on the color and chemical composition of the breast, thigh, and drumstick meat of crossbred Muscovy ducks

In this study, all ducks ate commercial concentrate as the feeding protocol from the farmers and drank saline water at different levels as an experimental design. This is the real situation at the farm level. However, our study did not analyze NaCl content in feed, which may influence protein or fat content in meat ducks. The red color of fresh meat is a critical factor that influences customer acceptance and selection. Some studies have found a relationship between muscle pH and meat color, indicating that lower muscle pH is associated with higher L* values, which represent lightness [26]. Moreover, previous research has shown that 24 h after slaughter, a decrease in beef pH is accompanied by an increase in the a* value, which represents redness [27]. However, this study did not confirm this relationship because lower pH in the thigh and drumstick did not result in changes in L* or a* values across treatments. The yellowness of meat, as indicated by the b* value, is more influenced by carotenoid intake; animals consuming large amounts of carotenoids tend to have more intense yellow coloration in their meat [28]. Since the ducks were fed the same diet in this study, the b* value remained consistent across treatments.

The protein content in the thigh and drumstick meat of crossbred Muscovy ducks was influenced by saline water, with higher salinity decreasing protein content. This reduction in CP content can be attributed to the lower CP intake observed in the present study. De Oliveira Carvalho *et al*. [21] reported that in slow-growing chickens, meat protein content increased with low levels of saline water but decreased at higher levels. In addition, although this study did not find any effect of saline water on fat content, it was observed that increasing salinity reduced fat content in other studies [21]. In this study, the protein content in Muscovy duck meat ranged from 18.6% to 20.8%, and the fat content ranged from 2.95% to 4.18%, which is consistent with the findings of da Silva Costa *et al*. [29].

### Effects of saline water on plasma biochemical parameters of crossbred Muscovy ducks

Muscovy ducks consuming saline water with salinity levels of 0.3%–0.6% showed increased plasma sodium and chloride levels, which is attributed to the high mineral content in the saline water used in this study. Similar results have been observed in ostriches [30], ducks, and gulls [31]. Ducks consuming fresh water had plasma electrolyte concentrations within the normal range, consistent with those reported by Park *et al*. [32]. High salinity in drinking water may damage muscle or liver enzyme activities in ducks, affecting kidney function and leading to elevated plasma concentrations of urea, creatinine, and AST, as noted by Bueno *et al*. [33] and Nasr *et al*. [34]. However, at low levels of saline water, liver enzyme function was not impaired throughout the experiment.

### Effects of saline water on the salt gland characteristics of crossbred Muscovy ducks

The consumption of saline water influences the salt gland characteristics of crossbred Muscovy ducks, which play a crucial role in osmoregulation by excreting excess salt. As the salinity levels in water increase, the salt glands of ducks may undergo adaptive changes, such as hypertrophy, to enhance their ability to excrete higher amounts of sodium and chloride [22]. These changes help ducks manage the increased salt load and maintain electrolyte balance. Laverty and Skadhauge [35] suggested that marine birds use post-renal transport mechanisms to eliminate excess NaCl and conserve water, in which the salt gland plays the major role. The size and weight of the salt glands typically correspond to the concentration of saline water consumed, with more pronounced effects observed at higher salinity levels. Similar findings were reported by previous studies of Hughes *et al*. [22] and Ali and Reshag [36], which observed that ducks consuming saline water had larger and heavier salt glands than those consuming fresh water. These adaptations are essential for the survival and well-being of ducks in environments with elevated salinity, although prolonged exposure to high salinity levels may lead to physiological stress or other health concerns.

## CONCLUSION

This study investigated the effects of saline drinking water on growth performance, carcass traits, blood biochemistry, and salt gland characteristics in crossbred Muscovy ducks. The findings revealed that increasing water salinity significantly reduced DMI and weight gain, particularly in male ducks. While male ducks exhibited a decline in final BW at salinity levels above 0.3%, female ducks maintained stable BWs up to 0.6% salinity. The FCR increased at higher salinity levels, indicating reduced feed efficiency. Carcass weight, breast weight, and abdominal fat percentage were negatively affected by saline water, while thigh and drumstick weights remained unchanged. In addition, saline water consumption altered blood biochemical parameters, with elevated plasma sodium, chloride, urea, and creatinine levels suggesting potential physiological stress. Notably, ducks in high salinity treatments developed larger salt glands, indicating an adaptive mechanism for salt excretion.

This study provides novel insights into sex-based differences in salinity tolerance among crossbred Muscovy ducks, which is valuable for farmers in saline-affected regions. The comprehensive evaluation of physiological and performance parameters, including growth, feed efficiency, meat quality, blood biochemistry, and salt gland morphology, offers a holistic understanding of the effects of saline drinking water. These findings have practical implications, highlighting the feasibility of farming female Muscovy ducks in saline-prone areas, which may help optimize livestock management in regions affected by climate change and saline intrusion.

Despite these contributions, certain limitations should be acknowledged. The study was conducted over a relatively short period, limiting the ability to assess long-term physiological and reproductive impacts of prolonged exposure to saline water. In addition, NaCl content in the feed was not analyzed, which could have influenced the observed effects on blood biochemistry and meat composition. While blood biochemical analysis suggested potential stress, further investigation into oxidative stress markers or endocrine responses would provide a more comprehensive assessment of physiological stress.

Future research should explore the long-term effects of saline water consumption on reproductive performance, immune function, and overall lifespan of Muscovy ducks. Investigating dietary interventions, such as supplementation with vitamin E, selenium, or osmoprotectants, may help mitigate the adverse effects of saline water on duck health and performance. Further studies on genetic and physiological adaptations in different duck breeds could identify those with enhanced salinity tolerance. Conducting field-based studies to validate the laboratory findings under real-world farming conditions, particularly in coastal regions affected by saline intrusion, would strengthen the practical applicability of these results.

This study underscores the differential tolerance of male and female Muscovy ducks to saline drinking water and provides practical recommendations for sustainable duck farming in saline-affected regions. While female ducks show superior adaptability, further research is needed to optimize management strategies and improve resilience against salinity-induced stress in poultry production.

## AUTHORS’ CONTRIBUTIONS

LTP, NTT, and NT: Conceptualized and designed the study. LTP and NT: Performed the animal experiments. NTT and NT: Performed the statistical analysis and interpreted the data. NT: Drafted and revised the manuscript. All authors have read and approved the final manuscript.

## ACKNOWLEDGMENTS

The authors are thankful to the manager of the Experimental farm at the College of Rural Development, Can Tho University, for supplying all the experiment materials and express sincere gratitude to Mr. Nguyen and Thuan for taking care of the experiment. The authors did not receive any funds for this study.

## COMPETING INTERESTS

The authors declare that they have no competing interests.

## PUBLISHER’S NOTE

Veterinary World remains neutral with regard to jurisdictional claims in published institutional affiliation.

## References

[1] Jalaludeen A, Churchil R.R, Baéza E (2022). Duck production and management strategies. Springer Nature, Singapore.

[2] Krista L.M, Carlson C.W, Olson O.E (1961). Some effects of saline waters on chicks, laying hens, poults, and ducklings. Poult. Sci.

[3] Tulu D, Hundessa F, Gadissa S, Temesgen T (2024). Review on the influence of water quality on livestock production in the era of climate change: Perspectives from dryland regions. Cogent Food Agric.

[4] Sulaiman A, Rahmatullah S, Effendi H, Simanungkalit G (2022). Production performance of Alabio ducks *(Anas platyrhynchos*Borneo) under different levels of drinking water salinity. J. Adv. Vet. Anim. Res.

[5] Anh N.N (2017). Historic drought and salinity intrusion in the Mekong River Delta in 2016: Lessons learned and response solutions. Vietnam J. Sci. Technol. Eng.

[6] Arias-Sosa L.A, Rojas A.L (2021). A review on the productive potential of the muscovy duck. J. Worlds Poult. Sci.

[7] Hughes M.R, Kojwang D, Zenteno-Savin T (1992). Effects of caecal ligation and saline acclimation on plasma concentration and organ mass in male and female Pekin ducks *Anas platyrhynchos*. J. Comp. Physiol. B.

[8] Association of Official Analytical Chemists (AOAC) (1990) Official Method of Analysis

[9] Zhang Y, Qi S, Fan S, Jin Z, Bao Q, Zhang Y, Zhang Y, Xu Q, Chen G (2024). Comparison of growth performance, meat quality, and blood biochemical indexes of Yangzhou goose under different feeding patterns. Poult. Sci.

[10] Anh V.T.L (2020). Some Biological Characteristics and Productivity of the Sea Ducks 15-Dai Xuyen Reared in Fresh and Saline Water Condition. Ph.D. Thesis.

[11] Khalilipour G, Maheri-Sis N, Shaddel-Teli A (2019). Effects of saline drinking water on carcass characteristics and litter moisture content of Japanese quails (*Coturnix coturnix Japonica*). Egypt J. Vet. Sci.

[12] Geerling J.C, Loewy A.D (2008). Central regulation of sodium appetite. Exp. Physiol.

[13] Odoi F.N.A, Afutu M.K, Lamptey S (2009). Effect of different salinity levels in drinking water on growth of broiler chickens. Ghana J. Agric. Sci.

[14] Sabat P (2000). Birds in marine and saline environments: Living in dry habitats. Rev. Chil. Hist. Nat.

[15] Chandrashekar J, Kuhn C, Oka Y, Yarmolinsky D.A, Hummler E, Ryba N.J.P, Zuker C.S (2010). The cells and peripheral representation of sodium taste in mice. Nature.

[16] Honarbakhsh S.S, Zaghari M, Shivazad M (2007). Can exogenous betaine be an effective osmolyte in broiler chicks under water salinity stress. Asian Austral. J. Anim. Sci.

[17] Goldstein D.L, Skadhauge E, Whittow G.C (2000). Renal and extra-renal regulation of body fluid composition. Sturkie's Avian Physiology.

[18] Ahmed A.S (2013). Performance and immune response of broiler chicks as affected by different levels of total dissolved solids in drinking water under hot arid environments. Anim. Prod. Sci.

[19] Hughes M.R (1989). Extracellular fluid volume and the initiation of salt gland secretion in ducks and gulls. Can. J. Zool.

[20] Hughes M.R (1995). Responses of gull kidneys and salt glands to NaCl loading. Can. J. Physiol. Pharmacol.

[21] De Oliveira Carvalho D.C, Gois G.C, da Silva L.C.C, de Souza F.F.N, Queiroz M.A.Á, Antunes K.V, de Souza Rodrigues R.T, Junior R.G.C.S, de Cássia Rodrigues, de Souza R, Pinheiro S.R.F, Neto A.F (2023). Effect of different levels of sodium in water on performance, carcass yield, and meat quality of slow-growing chickens. Trop. Anim. Health Prod.

[22] Hughes M.R, Bennett D.C, Sullivan T.M (2002). Effects of saline drinking water and water content of the gut and other organs of male and female mallards. Can. J. Zool.

[23] Marakan A, Galal A, El-Attar A.H (2017). Carcass parameters of Sudani, Muscovy duck strains and their cross. Egyptian J. Anim. Prod.

[24] Costa V.R, Cruz F.G.G, Rufino J.P.F, Silva A.F, Freitas B.K.M, Feijó J.C, Guimarães C.C (2019). Available phosphorus levels in diets for Muscovy ducks in housing. Braz. J. Poult. Sci.

[25] Kokoszyński D, Arpášová H, Hrnčar C, Żochowska–Kujawska J, Kotowicz M, Sobczak M (2020). Carcass characteristics, chemical composition, physicochemical properties, texture, and microstructure of meat from spent Pekin ducks. Poult. Sci.

[26] Da Silva D.C.F, de Arruda A.M.V, Gonçalves A.A (2017). Quality characteristics of broiler chicken meat from free-range and industrial poultry system for the consumers. J. Food Sci. Technol.

[27] Zhang Y, Hopkins D.L, Zhao X, van de Ven R, Mao Y, Zhu L, Han G, Luo X (2018). Characterisation of pH decline and meat color development of beef carcasses during the early postmortem period in a Chinese beef cattle abattoir. J. Integr. Agric.

[28] Umagiliya M.D, Bandara N, Jayasena D.D, Macelline S.P, Nawarathne S.R, Manjula P (2022). Comparison of meat quality traits in muscovy ducks reared under two different management systems. Anim. Ind. Technol.

[29] Da Silva Costa J, dos Santos W.M, Lemos I.M.T, dos Santos Braga B.S, dos Santos M.A.S, de Araújo Guimarães D.A (2023). Nutritional aspects and commercial challenges of Muscovy duck meat (*Cairina moschata*). Worlds Poult. Sci. J.

[30] El-Badry A.S.O, Ali A.A, Ali W.AA, Ahmed M.A (2011). The role of nasal gland and vitamin C in alleviate the adverse effects of osmotic stress on ostrich. Egypt. Poult. Sci.

[31] Roberts J.R, Hughes M.R (1984). Exchangeable sodium pool size and sodium turnover in freshwater-and saltwater acclimated ducks and gulls. Can. J. Zool.

[32] Park B.S, Um K.H, Park S.O, Zammit V.A (2018). Effect of stocking density on behavioral traits, blood biochemical parameters and immune responses in meat ducks exposed to heat stress. Arch. Anim. Breed.

[33] Bueno J.P.R, de Nascimento M.R.B.M, da Martins J.M.S, Marchini C.F.P, Gotardo L.R.M, de Sousa G.M.R, Mundim A.V, Guimarães E.C, Rinaldi F.P (2017). Effect of age and cyclical heat stress on the serum biochemical profile of broiler chickens. Semin Ciênc. Agrár.

[34] Nasr M.A.F, Alkhedaide A.Q, Radwan M.M.E, Hafez A.S.E, Hussein M.A, El Bayomi R.M (2022). Growth, carcass parameters, biochemical and oxidative stress indices, and meat traits of duck breeds under different stocking densities. Poult. Sci.

[35] Laverty G, Skadhauge E (2008). Adaptive strategies for post-renal handling of urine in birds. Comp. Biochem. Physiol. A Mol. Integr. Physiol.

[36] Ali M.A, Reshag A.F (2021). Morphometrical and histological changes in domestic duck salt glands in response to salinated water. Plant Arch.

